# Ear care: Knowledge, behavior, and attitudes among healthcare practitioners in Najran City, Saudi Arabia

**DOI:** 10.1371/journal.pone.0303761

**Published:** 2024-06-28

**Authors:** Ahmad Zaker M. Almagribi

**Affiliations:** Department of Surgery, College of Medicine, Najran University, Najran, Saudi Arabia; North South University, BANGLADESH

## Abstract

**Background:**

Personal care for body organs is a well-known practice of human beings, especially those organs that need regular care to improve function or hygiene. The ear is a unique sense organ with a specific anatomical shape to perform the function of hearing and balance.

**Objectives:**

To determine healthcare practitioners’ current knowledge, behavior, and attitude regarding ear care.

**Subjects and methods:**

This cross-sectional study was conducted among healthcare practitioners at different hospitals in Najran City, Saudi Arabia, from 25th June to 30th August 2022. A self-administered questionnaire was distributed among healthcare practitioners using an online survey. The questionnaire includes basic demographic characteristics (i.e. gender, speciality, and religion). It assesses the knowledge, behavior, and attitude toward ear care, and the use of mobile headphones and earrings that affect ear health. All statistical data were analyzed using SPSS version 26.

**Results:**

Of the 209 healthcare practitioners involved, 60.8% were females, and 46.9% were physicians. The prevalence of self-ear cleaning was 97.6%. Of them, 33% were cleaning their ears every week. Cotton buds were the most preferred method for self-ear cleaning. The proportion of participants who injured their ears while cleaning was 8.6%. The most common treatment method to relieve ear pain was visiting a doctor (44.4%) and utilizing a painkiller (29.7%). Interestingly, respondents who injured their ears during cleaning and those who experienced wax accumulation were significantly more common among physicians.

**Conclusion:**

Self-ear cleaning practices are widely prevalent in this study which could be related to the lack of knowledge about ear care among healthcare practitioners. Physicians who experienced wax accumulation tend to use other methods for self-ear cleaning rather than cotton buds. Further research is needed to determine the knowledge, attitude, and practices of the population who are working in healthcare institutions.

## 1. Introduction

The human ear is a prominent and often-referenced bodily feature. Due to its importance in the hearing process, maintaining good ear health is a priority for everyone. Maintaining clean ears is an example of good social hygiene. Although it does not require a lot of maintenance, it will perform better if you give it some attention. Ear hygiene can be accomplished in a number of different ways. The best approach to keep your ears healthy is to take care of them [1]. Other measures include keeping them clean, keeping them away from loud environments, shielding them from harm, and avoiding getting them damaged in any manner.

Getting rid of accumulated earwax is a crucial part of maintaining clean ears. Cerumen, often known as earwax, is a waxy substance that is naturally produced by ceruminous and sebaceous glands in the ear canal’s external auditory meatus. Ears need a certain amount of earwax to function properly [2]. The "conveyor belt" process of epithelial migration in a healthy external canal is self-cleaning, with the help of jaw movement [3]. This method is used to clean the ear canal and remove debris like cerumen and dust. Incorrect ear cleaning techniques can lead to cerumen impaction, otitis externa, and even damage [4].

Cotton swabs and hairpins are common tools for removing ear wax, however they can cause permanent damage to the ear canal and are best avoided. The use of these tools is discouraged because of the potential damage they can do to your hearing. Loss of hearing may result from prolonged exposure to loud noises and loud construction sites. Hearing loss can be caused by prolonged exposure to loud noises, such as those produced by mobile phones or music players [2].

Cerumen blockage, also known as cerumen impaction, blocks the ear canal and results in ear pain, itching, and hearing loss [5]. Cerumen makes it difficult for the doctor to properly assess the inner ear and audiovestibular system, thus the doctor may need to clear out the earwax [6,7]. Self-ear cleaning is the practice of many people who put various things into their ears to eliminate earwax. They believe that removing extra earwax is crucial for maintaining excellent ear hygiene [8,9] and that cerumen is a sign of an infection or that its impaction bothers and hurts them [10,11]. Unsafe methods of removing earwax, such as using cotton buds or matchsticks, can result in injuries [12–14], otitis cerumen impaction, or otitis externa, which has a significant risk of hearing loss. By informing the public on the risks associated with ear self-cleaning, we can avoid or lessen injuries and symptoms related to ear self-cleaning [8].

Although medical students are generally thought to have a higher level of knowledge than the general public, a small percentage of preclinical students showed inadequate knowledge, attitude, and practice when it came to ear care [15]. In 2015, researchers in Nigeria conducted another survey and found that 92.8% of the participants used cotton swabs to clean their ears. One of the most prevalent applications of cotton swabs is to soothe itchy ears. Incorrectly believing that cotton swabs are helpful, they have a severely skewed understanding of the topic. Ear cleaning is not something that doctors typically recommend. Public health and education should be prioritized to increase people’s understanding of the issue [16].

In Saudi Arabia, specifically in Najran City, the knowledge, behavior, and attitudes of healthcare practitioners regarding ear care is not previously explored. Understanding the current practices and perceptions among healthcare practitioners can shed light on the effectiveness of existing educational programs and identify areas that require improvement. Furthermore, healthcare practitioners themselves serve as role models for patients and the community, making their knowledge and behavior crucial in promoting good ear care practices.

This study aims to assess the knowledge, behavior, and attitudes of healthcare practitioners in Najran City, Saudi Arabia, regarding ear care. By examining their practices, including self-ear cleaning methods and frequency, as well as their attitudes towards the use of mobile headphones and earrings, this research seeks to identify potential gaps in knowledge and areas where further education and awareness campaigns are needed.

The prevalence of self-ear cleaning, choice of cleaning methods, and the occurrence of ear injuries among healthcare practitioners will be explored. Additionally, the study will investigate the preferred treatment methods adopted by healthcare practitioners to alleviate ear pain. By considering demographic characteristics such as gender, specialty, and religion, the research aims to identify any variations or trends that may exist within the healthcare practitioner population.

The rationale for conducting this study lies in the significance of ear care, the role of healthcare practitioners in promoting it, and the need to assess their knowledge, behavior, and attitudes. By addressing the gaps in understanding and identifying areas for improvement, this research aims to contribute to the advancement of ear care practices and outcomes in Najran City, Saudi Arabia.

The findings of this study will provide valuable insights into the current state of ear care knowledge, behavior, and attitudes among healthcare practitioners in Najran City. It will help identify areas for improvement in educational programs and interventions targeting healthcare practitioners, with the ultimate goal of enhancing the quality of ear care provided to patients and the wider community in Saudi Arabia and beyond.

## 2. Methods

### 2.1. Study population and sample size calculation

This is a cross-sectional study conducted from 25^th^ June to 30^th^ August 2022, among healthcare practitioners at different hospitals in Najran City, Saudi Arabia. ***Sample Size***: The total number of HCPs in these hospitals was approximately 900. A minimum sample size of 209 was calculated using the Raosoft sample size calculator (http://www.raosoft.com/samplesize.html, retrieved on June 15, 2022) using the 90% confidence level, a 5% margin of error, and a 50% sample percentage. ***Sampling technique***: The healthcare practitioners employed at several hospitals in Najran City, Saudi Arabia were recruited through the utilization of simple random sampling technique. A total of 300 healthcare practitioners from 18 hospitals were reached out to, out of which 209 provided positive responses and agreed to take part in the study, resulting in a response rate of 69.66%.

### 2.2. Study tool

A self-administered questionnaire was distributed among healthcare practitioners using an online survey. Data was gathered through the use of a self-administered questionnaire. Existing literature was reviewed extensively to guide the development of the questionnaire (2–11). The questionnaire includes basic demographic characteristics (i.e. gender, specialty, and religion), a questionnaire that assesses the knowledge, behavior, and attitude toward ear care and the practice toward the use of mobile headphones, and earrings that affects ear health. The research equipment went through extensive adaptation to make sure it was a good fit for the traits of the subjects. Using social media platforms like Twitter, LinkedIn, Facebook, and WhatsApp status, we distributed the questionnaire link.

### 2.3. Validity and reliability of study tools

The preliminary form of the questionnaire was reviewed and examined by specialists from the departments of family and community medicine and epidemiology at Najran University to ensure its accuracy. To ensure uniformity, the questionnaire was first translated into Arabic by a professional translator who was impartial, and then it was translated again into English by a second professional translator. Twenty people took part in a pilot study to evaluate the validity and reliability of the survey. The final count included the subjects’ outcomes from the pilot research. In addition, the reliability of each question on the questionnaire was assessed, and the results showed that Cronbach’s alpha factor was 0.85, indicating a good level of internal consistency.

#### Exclusion criteria

The exclusion criteria included non-healthcare practitioners, healthcare practitioners located outside Najran City, participants who provide inaccurate or unreliable responses, and those who are unable to provide informed consent. The inclusion of these criteria guarantees that the study concentrates on healthcare practitioners who are pertinent to the specified region, preserves the precision and dependability of the data, and adheres to ethical principles.

### 2.4. Ethical considerations

All procedures followed the guidelines laid down in the Declaration of Helsinki, and the study was given the go-ahead by the Najran University’s Ethics Committee (443-42-68856-DS). All participants were given an informed consent form and their written consent was obtained. The questionnaire was constructed to ensure the confidentiality of all responses. After this, participants were guaranteed that their confidentiality would be maintained when the study results will be released.

### 2.5. Statistical analysis

Descriptive statistics were summarized as numbers and percentages (%). The knowledge, behavior, and attitude between physicians and non-physicians toward ear care were calculated by using the Chi-square test. A *P*-value of 0.05 was considered statistically significant. The data were analyzed using Statistical Packages for Social Sciences (SPSS) version 26 (Armonk, NY: IBM Corp, USA).

## 3. Results

This study involved 209 healthcare practitioners. As described in Table 1, 46.9% were doctors with the majority being females (60.8%) and Muslim religion constitute most of the respondents (76.6%).

**Table 1:** Basic demographic characteristics of the healthcare practitioners (N = 209).

Study variables	n (%)
*Specialty*	
Doctor	98 (46.9)
Nurse	76 (36.4)
Technician	14 (06.7)
Other allied healthcare practitioners	21 (10.0)
*Gender*	
Male	82 (39.2)
Female	127 (60.8)
*Religion*	
Muslim	160 (76.6)
Non-Muslim	49 (23.4)

Values are presented as numbers and percentages (%).

Regarding healthcare practitioners’ knowledge, behavior, and attitude toward ear care (Table 2), one-third (33%) of them were cleaning their ears on weekly basis with cotton buds as the most prominent tool to clean their ears. The proportion of the respondents who injured their ears while cleaning was 8.6%. Of them, external auditory canal abrasion was identified as the most common type of injury that happened to the ear (50%) wherein 72.2% expressed that it improved spontaneously. The prevalence of participants who experienced wax accumulation was 40.2% wherein 63.1% reported the use of cotton buds to remove it while 22.6% complained of abnormal ear sounds due to wax accumulation.

**Table 2:** Knowledge, behavior, and attitude toward ear care (N = 209).

Statement	n (%)
*How frequently do you clean your ears*?	
Never	05 (02.4)
Occasionally	67 (32.1)
Weekly	69 (33.0)
1 to 4 times daily	21 (10.0)
5 times or more daily	47 (22.5)
*How did you clean your ears usually*?	
With cotton buds	159 (76.1)
With fingers	29 (13.9)
Car keys	02 (01.0)
Pens cover	02 (01.0)
Tip of eyeglass	02 (01.0)
Others	15 (07.2)
*Did you ever injure your ears during cleaning*?	
Yes	18 (08.6)
No	191 (91.4)
*What was the type of injury to the ear*? ^*(n = 18)*^	
External auditory canal abrasion	09 (50.0)
Tympanic membrane perforation	02 (11.1)
Foreign body impaction	03 (16.7)
Bleeding	01 (05.6)
Others	03 (16.7)
*What did you do to treat this injury*? ^*(n = 18)*^	
Healed spontaneously	13 (72.2)
Visit specialized clinic	04 (22.2)
Visit Pharmacy	01 (05.6)
*Did you ever experience wax accumulation at any time*?	
Yes	84 (40.2)
No	125 (59.8)
*What did you do to remove it*? ^*(n = 84)*^	
Cotton buds	53 (63.1)
Visit specialized clinic	20 (23.8)
Olive oil	02 (02.4)
Visit Pharmacy	02 (02.4)
Others	07 (08.3)
*What was the complaint*? ^*(n = 84)*^	
Hearing loss	13 (15.5)
Abnormal ear sound	19 (22.6)
Irritation	12 (14.3)
Nothing	40 (47.6)

Values are presented as numbers and percentages (%).

In Table 3, approximately 38.8% used mobile headphones regularly. Of them, 32.3% complained about ear pain with 24.8% used to connecting it to a loud sound, whilst 10.5% ask for medical help due to persisting symptoms. The proportion of respondents who wear earrings was 45%. Of them, 7.3% experienced complications, with 40.6% done ear piercings during childhood, however, 86.5% were regularly cleaning the site of the rings. The prevalence of respondents who experienced ear problems during air travel was 35.9%, while those who experienced an ear problem after using medication were 2.4%. Of those who experienced ear problems after taking medication (n = 5), 2 cases experienced ear pain and another 2 cases experienced ear fullness with most of these patients visiting a physician for treatment, whereas, 2 cases received gentamycin as medication.

**Table 3:** Practices toward the use of mobile headphones, earrings that affects ear health (N = 209).

Statement	n (%)
*Did you use mobile headphones usually*?	
Yes	107 (51.2)
No	102 (48.8)
*Do you feel any of these complaints after using mobile headphones*? ^*(n = 133)*^	
Ear pain	43 (32.3)
Decrease hearing	10 (07.5)
Abnormal sound in the ear	09 (06.8)
Nothing	71 (53.4)
*Did you connect it to a loud sound usually*? ^*(n = 133)*^	
Yes	33 (24.8)
No	65 (48.9)
Maybe	35 (26.3)
*Does that symptom ever need medical help*? ^*(n = 133)*^	
Yes	14 (10.5)
No	119 (89.5)
*Did you wear earrings*?	
Yes	94 (45.0)
No	113 (54.1)
Multiple	02 (01.0)
*Did you experience any complications after piercing*? ^*(n = 96)*^	
Yes	07 (07.3)
No	89 (92.7)
*How did you do ear piercing*? ^*(n = 96)*^	
Personally	08 (08.3)
At home by another person	10 (10.4)
In childhood by family	39 (40.6)
Traditional expert	09 (09.4)
At doctor clinic	27 (28.1)
Others	03 (03.1)
*Do you clean the site of rings*? ^*(n = 96)*^	
Yes	83 (86.5)
No	06 (06.3)
Maybe	07 (07.3)
*Did you experience any ear problems during air travel*?	
Yes	89 (42.6)
No	120 (57.4)
*Have you ever experienced an ear problem after using any medication*?	
Yes	05 (02.4)
No	204 (97.6)
*What was the complaint*? ^*(n = 5)*^	
Ear pain	02 (40.0)
Ear fullness	02 (40.0)
Dizziness	01 (20.0)
*How this manifestation was relieved*? ^*(n = 5)*^	
Visit physician	03 (60.0)
Valsalva maneuver	01 (20.0)
Others	01 (20.0)
*What was the medication*? ^*(n = 5)*^	
Gentamycin	02 (40.0)
Others	03 (60.0)

Values are presented as numbers and percentages (%).

**Fig 1:**
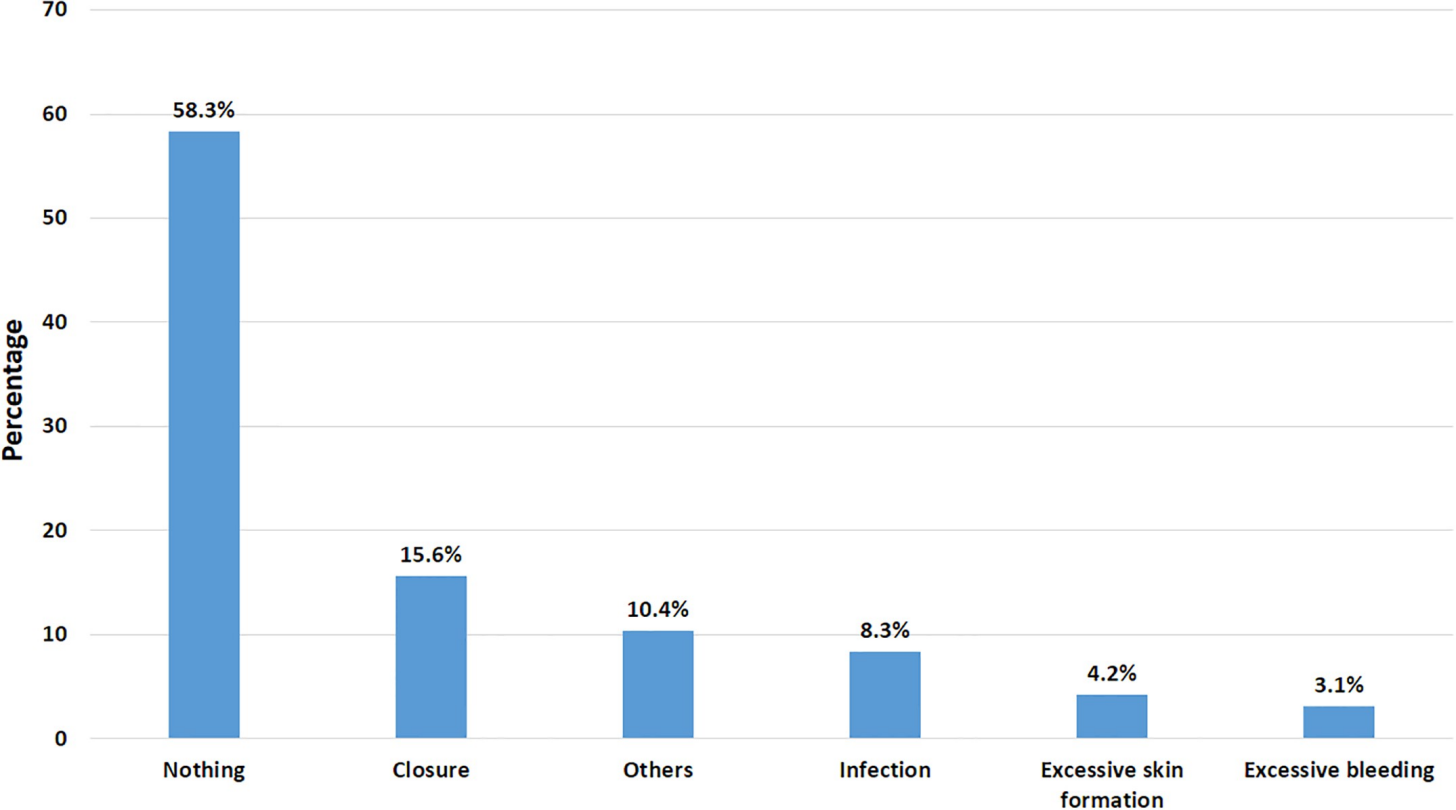
Complication after ear piercing.

In Fig 1 the common complications sustained by healthcare practitioners after ear piercing was closure (15.6%), and infection (8.3%).

**Fig 2:**
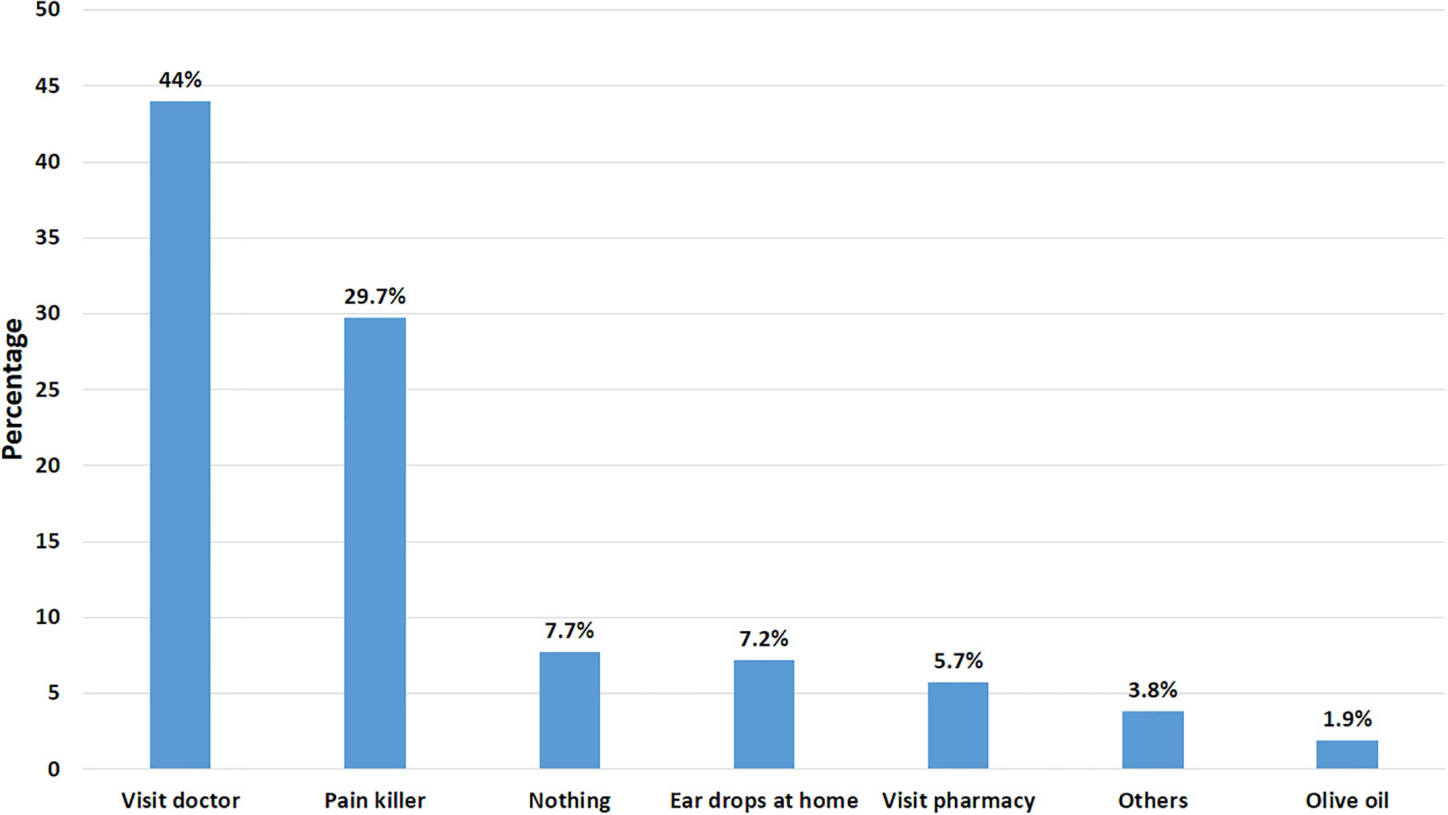
Treatment method to relieve ear pain.

In Fig 2 the common treatment method to relieve ear pain was visiting a doctor (44%), followed by pain killer (29.7%) and ear drops at home (7.2%).

**Fig 3:**
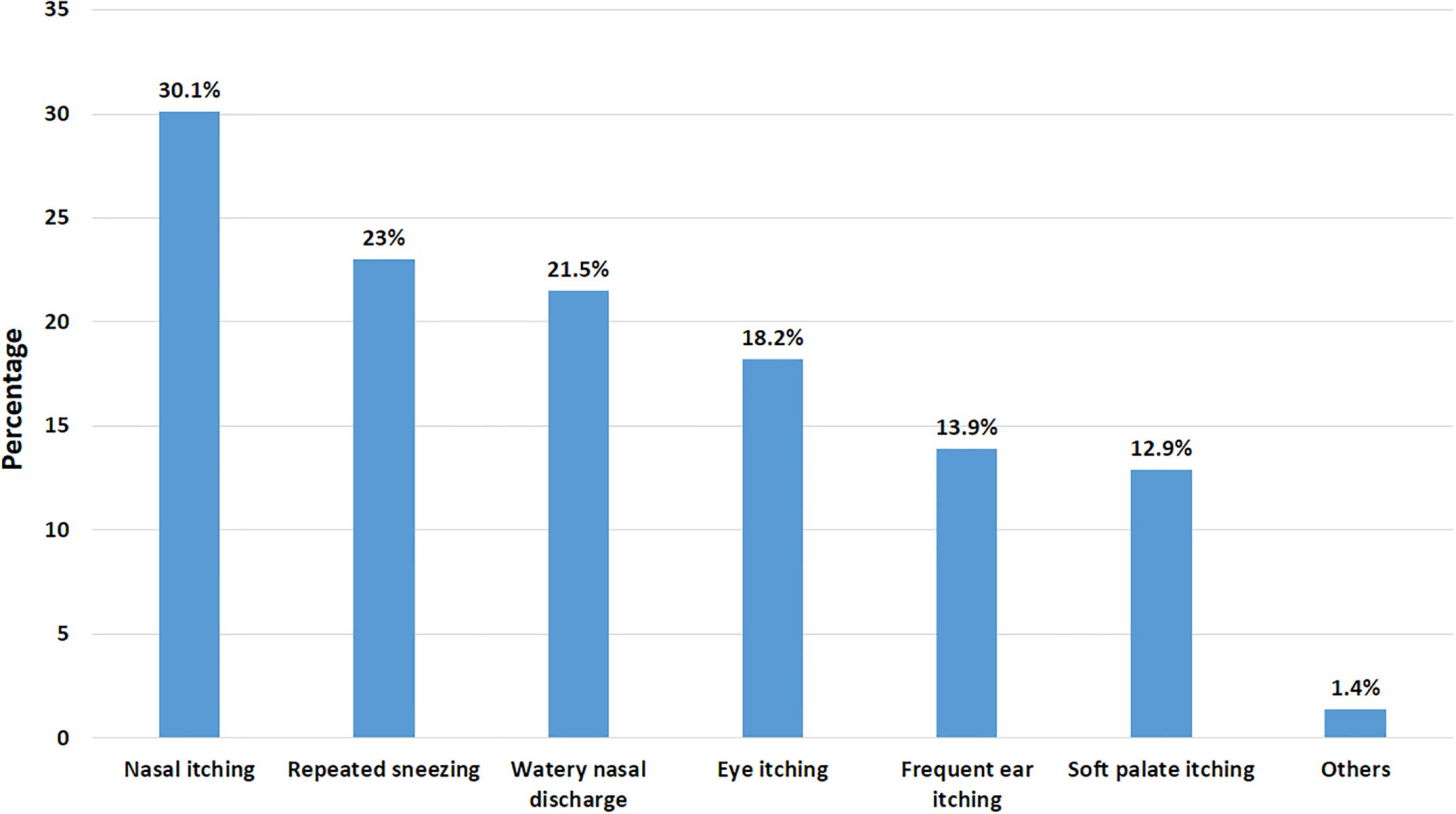
Signs and symptoms of allergy.

In Fig 3 the most common sign and symptom of allergy was nasal itching (30.1%), followed by repeated sneezing (23%) and watery nasal discharge (21.5%).

In Table 4. it was observed that physicians were more associated with having an incidence of injured ears during cleaning (p = 0.024), experiencing wax accumulation (p = 0.017), knowing signs and symptoms of allergy such as watery nasal discharge (p = 0.001), soft palate itching (p = 0.001) and frequent ear itching (p = 0.003) while non-physicians were more associated with using cotton buds for ear cleaning (p<0.001) and for wearing earrings (p<0.001).

**Table 4:** Knowledge of the signs and symptoms of allergy, experienced ear complications, and practice of ear care between physicians and non-physicians (N = 209).

Factor	Healthcare practitioners		P-value ^§^
	Physicians n (%)^(n = 98)^	Non-physicians n (%)^(n = 111)^	
*Frequency of ear cleaning*			
1 to more than 5 times daily	28 (28.6)	40 (36.0)	0.187
Weekly	30 (30.6)	39 (35.1)	
Occasionally	40 (40.8)	32 (28.8)	
*Method of ear cleaning*			
With cotton buds	61 (62.2)	98 (88.3)	**<0.001** **
Other methods	37 (37.8)	13 (11.7)	
*Injured ears during cleaning*			
Yes	13 (13.3)	05 (04.5)	**0.024** **
No	85 (86.7)	106 (95.5)	
*Experienced wax accumulation*			
Yes	48 (49.0)	36 (32.4)	**0.017** **
No	50 (51.0)	75 (67.6)	
*Usually used mobile headphones*			
Yes	45 (45.9%)	62 (55.9%)	0.151
No	53 (54.1%)	49 (44.1%)	
*Wear earrings*			
Yes	30 (30.6)	66 (59.5)	**<0.001** **
No	68 (69.4)	45 (40.5)	
*Experienced any ear problems during air travel*			
Yes	45 (45.9%)	44 (39.6%)	0.360
No	53 (54.1%)	67 (60.4%)	
*Signs and symptoms of allergy* *			
Nasal itching	36 (36.7)	27 (24.3)	0.051
Repeated sneezing	24 (24.5)	24 (21.6)	0.623
Watery nasal discharge	31 (31.6)	14 (12.6)	**0.001** **
Eye itching	21 (21.4)	17 (15.3)	0.284
Soft palate itching	21 (21.4)	06 (05.4)	**0.001** **
Frequent ear itching	21 (21.4)	08 (07.2)	**0.003** **
Others	03 (03.1)	0	0.063

Values are presented as numbers and percentages (%).

* Variables with multiple response answers.

^§^ P-value has been calculated using the Chi-square test.

** Significant at p<0.05 level.

## 4. Discussion

The present study is conducted to find out the current knowledge, behavior, and attitude of healthcare practitioners regarding ear care. The findings of this study revealed that most of the study population were doing self-ear cleaning with an overall prevalence of 97.6%, ranging from occasionally (32.1%) to 5 times or more self-ear cleaning per day (22.5%). A high prevalence of self-ear cleaning had also been documented among healthcare professionals in Nigeria (1), with 91.5% practicing self-ear cleaning with the majority doing it occasionally (37.2%). This is almost consistent with the study conducted among the doctors in Northwestern Nigeria (2), wherein the prevalence of cotton bud users was 93.4% and was used at least once daily. In South Africa (3), most of the university students (98%) engaged in self-ear cleaning practices, suggesting that it is beneficial although 2.4% reported injury associated with self-ear cleaning. In Riyadh, a great proportion of medical students had good knowledge about self-ear cleaning behavior (4). Nearly two-thirds (65.5%) used to clean their own ears mostly with cotton buds (79%) to remove cerumen (30.8%). Among otorhinolaryngology patients (5), the prevalence was consistent at 93.4% with a little below half of them (49.3%) doing it on a daily basis.

A study conducted in Majmaa, Saudi Arabia, documented that 55.4% of the university students showed poor knowledge about the practices of self-ear cleaning (6). Contradicting this report, a study conducted in Jazan, Saudi Arabia, revealed that there were good knowledge (91.9%), attitude (90.4%), and practices (83.9%) among the general population toward self-ear cleaning [17]. Among the determinant of knowledge, 54% of their subjects do not believe that ear wax should be removed continuously, and neither their population had the habit of ear cleaning with sharp objects such as pencils, pins, and earbuds (81.6%) nor use of oil in ears (94.4%). In Abha, Saudi Arabia, more than half (55.1%) of the students thought self-ear cleaning is beneficial and approximately three-quarters were actually practicing it [18]. Also, nearly half were practicing it for more than 5 years. In this scenario, the authors noted that medical students exhibited higher knowledge and lower ear self-cleaning practices more than non-medical students did. It is surprising to know that our healthcare practitioners were practicing self-ear cleaning more frequently which is not recommended. Hence, continuous education is warranted to update healthcare practitioners’ information about ear care and correct their improper practices.

Even though most of our sample population use cotton buds for self-ear cleaning (76.1%), however, some of them use other methods including fingers (13.9%), and other methods (10.2%) such as car keys (1%), pens cover (1%) and a tip of the eyeglass (1%). Conversely, among those who experienced wax accumulation (40.2%), besides cotton buds (63.1%), nearly one quarter (23.8%) preferred to visit a specialized clinic while others use olive oil (2.4%) and OTC medications (2.4%) to remove it. Incidentally, only 8.6% reported injuring ears due to persistent self-ear cleaning with external auditory canal abrasion being the most common type of ear injury. In a study carried out among health workers in Nigeria, only 10.7% demonstrated good knowledge of ear wax and the health effects of self-ear cleaning while 51.3% had poor knowledge (9). The Nigerian study also cited that the most commonly reported complications of cerumen were ear itchiness (29.3%), ear infection (20.4%), earache (20%), deafness (17.3%), and making one feel dirty (13%). In Riyadh, researchers indicated that inappropriate practices in ear care were seen among the rural population in such a way that most of them use objects to clean the ear canal, sudden or gradual exposure to loud noise, use ear drops randomly, and blowing the nose roughly, adding that the knowledge regarding the harmful effect of using foreign or sharp objects was found to be lacking but was better in urban population (10). While in Makkah, Saudi Arabia, approximately half of the population (50.4%) believed that constant self-removal of ear wax is inappropriate, however, 32% were cleaning their ears to remove dirt while 29.3% were doing it to improve ear hygiene (11). Self-ear cleaning is a common practice that may not be able to prevent. Along with this, improper ear care practices are tantamount to some ear complications. Therefore, continuous health education is needed to rectify public views about self-ear cleaning and to prevent problems associated with incorrect self-ear cleaning practices.

The use of other tools or accessories can cause some complications to the ears. This perception is consistent with the beliefs of the people living in the Jazan region, Saudi Arabia (7). According to reports, 58.5% of the respondents believed that exposure to loud noise can cause deafness while 41.9% were actually being exposed to loud noise due to the use of the microphone head seat. Ear piercing was also mentioned by 55.8% as a must to be done during childhood or as early as after birth whereas most of the respondents believed that low and high altitudes can also cause ear pain. These findings are almost consistent with our results wherein nearly 40% of our respondents were mobile headphones users with 32.3% complaining of ear pain due to excessive use including listening to loud sounds (24.8%). Likewise, 45% were wearing earrings, though its complication was less (7.3%) which could be attributed to having good ear hygiene, wherein 86.5% expressed that they clean regularly the site of rings. In addition, 35.9% reported experiencing ear pains during the take-off or landing of an airplane. Although, our sample population exhibited knowledge in some aspects, however, their in-depth knowledge needs more improvement.

Moreover, we also evaluated the knowledge, behavior, and practices between physicians and non-physicians, and based on our results, we have learned that non-physicians were the significant users of cotton buds and were prevalent with wearing earrings. On the other hand, the incidence of ear injury and wax accumulation happened more frequently among physicians and they exhibited better knowledge about the signs and symptoms of allergy such as watery nasal discharge, soft palate itching, and frequent ear itching. In Northwestern Nigeria (2), they found that the use of cotton buds was significantly related according to the departments where the doctors were assigned. Conversely, citing a risk of possible harmful effects due to improper self-ear cleaning practices in Nigeria, the researchers found out that the prevalence of self-ear cleaning practices was significantly higher in gender females, those who believed the habit was beneficial, and those whose family and relatives practiced the habit (12). However, in India (13), 66.7% to 90% across different educational levels were not sure that ’cold’ can lead to ear infections, and 46.7% to 75% do not have enough information that diabetes and hypertension can reduce the sense of hearing, whereas, in Nigeria (14), most otorhinolaryngology patients wrongly believed that self-ear cleaning is beneficial which could be associated with an increased incidence of ear complications. Hence, external auditory canal injury (28.9%) and impacted foreign body (25.6%) were cited as the most commonly known complications that occurred to patients.

The findings of a recent study conducted in South Korea using structural equation modeling revealed a significant relationship between knowledge and the attitudes and health-seeking practices of allied healthcare professionals. This study demonstrated the substantial impact of knowledge on attitudes and health-seeking practices. This finding unequivocally demonstrates that a substantial level of knowledge serves as the primary motivator for the adoption of health-seeking behaviors and the cultivation of good attitudes among both facility managers and healthcare professionals. Therefore, we propose that an additional measure be taken, namely the establishment of comprehensive and professional guidelines pertaining to hearing care information for these professionals. We posit that such endeavors have the potential to enhance knowledge, attitudes, and practices related to hearing [19]

The use of conservative/medical treatment was widely prevalent (71.1%) among the Indian population (13). These findings almost mirrored our results. In our study, visiting a doctor was the most preferred treatment method by our population (44%), followed by painkiller medication (29.7%). Other preferred methods were ear drops at home (7.2%), OTC drugs (5.7%), and olive oil being the least preferred (1.9%). Additionally, we observed that external auditory canal abrasion (50%) foreign body impaction (16.7%), and tympanic membrane perforation were cited as the most commonly known type of injury that happened to the ear. More investigations are warranted to shed more light on the most common complication and the most preferred treatment method for ear complications necessary to improve ear care.

## 5. Conclusion

Self-ear cleaning practices are widely prevalent in this study which could be related to the lack of knowledge about ear care among healthcare practitioners. Physicians who experienced wax accumulation tend to use other methods for self-ear cleaning rather than cotton buds. The use of different objects in ears was reported by many although complications were minimal. Despite our sample population working in the medical field, still we found their knowledge of ear care unsatisfactory. Therefore, extensive efforts are needed to bridge the gaps in knowledge, behavior, and practices toward ear care. More courses and workshops are needed to update the information of our healthcare practitioners. As healthcare practitioners are the most reliable sources of information for the community, hence, their knowledge about ear care should be up to date so they can provide adequate and relevant information whenever someone sought to. Further research is needed to determine the knowledge, attitude, and practices of the population who are working in healthcare institutions.

## 6. Limitations

Several limitations are worth noting for this study. The study conducted was a cross-sectional investigation that included healthcare professionals (HCP) from multiple hospitals located in Najran city, Saudi Arabia. Therefore, the findings derived from this research are not applicable to the entire population of Saudi Arabia. In addition to this, the measurement of Ear care KAP was conducted through the participants’ self-reporting. Therefore, the accuracy of the data may be compromised as a result of either over-reporting or under-reporting.

## 7. Recommendations for healthcare practitioners to follow and share with their patients

The following are a few important points related to the implementation of safe and efficient ear care practices, accompanied with specific recommendations for healthcare professionals to follow and communicate to their patients:

1. **Avoid inserting objects into the ear canal.** It is crucial to highlight that the act of inserting objects such as cotton swabs, bobby pins, or similar items into the ear canal has the potential to exacerbate the accumulation of wax and potentially result in harm.
2. **Allow natural wax migration.** The auditory system possesses an inherent mechanism for self-cleansing, wherein wax spontaneously moves from the auditory canal to the external ear. Patients should be advised to allow the wax to naturally expel from the ear without any intervention.
3. **Utilize ear drops.** Non-prescription ear drops might facilitate the softening of wax, hence enhancing its natural expulsion. It is recommended that patients adhere to the directions provided on the packaging of the ear drops and seek guidance from a healthcare practitioner in the event of any concerns.
4. **Seek medical assistance when necessary.** If a patient encounters symptom such as intense ear discomfort, hearing impairment, chronic itching, or a sensation of ear fullness, it is recommended that they seek medical treatment. The presence of these symptoms may suggest the presence of an underlying condition that necessitates a professional assessment and intervention.
5. **Professional ear cleaning.** Healthcare practitioners can do professional ear cleaning when wax impaction is causing pain or hearing loss. The safe removal of affected wax may necessitate the utilization of specialist instruments or irrigation procedures. It is important to notify patients that professional cleaning should exclusively be performed by individuals who have received proper training.
6. **Provide written instructions.** In order to strengthen the knowledge imparted during consultations, healthcare professionals have the option to furnish written instructions or pamphlets pertaining to the implementation of safe and efficient ear care protocols. This document will function as a valuable resource for patients to adhere to while residing at home.
7. **Stay up to date with guidelines.** Healthcare practitioners should regularly update their knowledge of current guidelines and best practices regarding ear care. This will enable them to provide the most accurate and evidence-based recommendations to their patients.

By following these safe and effective ear care practices and sharing these recommendations with their patients, healthcare practitioners can contribute to promoting proper ear health and minimizing the risks associated with incorrect ear cleaning methods.

## 8. Future implications

The present study’s findings have significant implications for future ear care research. Future research should evaluate the efficacy of educational interventions for healthcare practitioners and the general public, assess long-term outcomes of safe ear care practices, understand patient behavior and decision-making regarding ear care, compare the effectiveness of different cleaning methods, conduct cost-effectiveness analyses, and investigate the specific needs of special populations and vulnerable groups. Addressing these study implications will help to promote adequate ear care, develop evidence-based interventions, and improve ear health outcomes for people from varied backgrounds.

## 9. Limitations

The current study has several limitations that should be acknowledged. Firstly, the study relies on self-reported data obtained through a questionnaire, which may be subject to recall bias or social desirability bias. Secondly, the study’s cross-sectional design provides a snapshot of the participants’ knowledge, behavior, and attitudes at a specific point in time. Thirdly, the study’s sample is limited to healthcare practitioners in Najran City, which may not be representative of other regions in Saudi Arabia or healthcare practitioners in different settings. Additionally, the study relies on self-administered questionnaires distributed online, which may introduce selection bias by excluding individuals who do not have internet access or are less likely to participate in online surveys.

## Supporting information

S1 Dataset (XLSX)
